# Sleep and the Developing Brain: What We Know, What We Don’t, and Where to Act—A Systematic Review and Evidence and Gap Map

**DOI:** 10.3390/ejihpe16080120

**Published:** 2026-08-20

**Authors:** Kuanysh Shonbay, Olga M. Shikhova, Yevgeniy Duplyakin, Raikhan S. Sagimova, Maiya Taushanova, Indira Karibayeva

**Affiliations:** 1 Higher School of Psychology, Turan University, Almaty 050013, Kazakhstan; k.shonbay@turan-edu.kz (K.S.); e.duplyakin@turan-edu.kz (Y.D.); 2 Department of Pediatrics, Asfendiyarov Kazakh National Medical University, Almaty 050012, Kazakhstan; raikhans09@gmail.com; 3 Department of Public Health and Healthcare with a Nursing Course, West Kazakhstan Marat Ospanov Medical University, Aktobe 030019, Kazakhstan; dr.taushanova@zkmu.kz; 4 Department of Research Management, JSC Research Institute of Cardiology and Internal Diseases, Almaty 050012, Kazakhstan; 5 Department of Health Policy and Community Health, Jiann-Ping Hsu College of Public Health, Georgia Southern University, Statesboro, GA 30458, USA

**Keywords:** sleep duration, sleep quality, sleep timing, chronotype, executive function, attention, emotion regulation, academic performance, children, adolescents, evidence and gap map, systematic review

## Abstract

Sleep supports cognition across the lifespan, but its significance may differ developmentally: in adults, it largely maintains and restores functioning within mature neural systems, whereas in children and adolescents it may also support the formation of cognitive, emotional, and self-regulatory capacities. This systematic review and evidence and gap map, prospectively registered in PROSPERO and conducted according to PRISMA 2020, searched PubMed, PsycINFO, Scopus, and Web of Science for studies of sleep duration, quality/fragmentation, timing/regularity, and daytime sleepiness in relation to executive function/self-regulation, attention/vigilance, emotion/behavior regulation, and academic functioning among typically developing children and adolescents. Twenty-five studies contributed 46 study–outcome links. Across nearly all populated evidence cells, more adequate sleep profiles were associated with better developmental outcomes. The strongest and most experimentally supported evidence concerned sleep duration in relation to executive function, emotion/behavior regulation, and academic functioning; evidence for sleep timing/regularity and daytime sleepiness was weaker and predominantly cross-sectional. Unlike adults, for whom sleep primarily restores a mature system, children may lose not only today’s functioning but tomorrow’s developmental trajectory when sleep is chronically curtailed. Sleep duration currently provides the strongest basis for intervention, whereas the long-term developmental effects of sleep quality, timing, regularity, and sleepiness require further longitudinal and experimental investigation during this formative period.

## 1. Introduction

Sleep is a multidimensional health behavior encompassing not only duration but also continuity, perceived quality, timing, regularity, and the degree of sleepiness experienced during waking hours. Each of these dimensions may have distinct implications for development and daily functioning. In children and adolescents, inadequate or disrupted sleep has been linked to poorer cognitive performance, difficulties with behavioral and emotional regulation, and less favorable educational outcomes (Liu et al., 2022). Longitudinal evidence further suggests that insufficient sleep in early adolescence may be associated with persistent differences in cognition, behavior, and brain development, although observational studies cannot establish causality (Yang et al., 2022). It is therefore important to differentiate among specific sleep parameters rather than treating sleep as a uniform exposure.

Recent reviews have addressed parts of this evidence base, but most have focused on a single sleep parameter, outcome group, or restricted population. For example, a systematic review of population-based studies on sleep duration found substantial variation in measurement approaches, cutoff points, and outcome definitions, and noted that studies of cognitive and academic outcomes in children and adolescents often had lower methodological quality (Machado et al., 2022). A meta-analysis of U.S. secondary-school students examined sleep duration and quality in relation to academic performance and reported different associations for the two dimensions (Musshafen et al., 2021). Similarly, a systematic review of sleep timing identified possible links with emotional regulation and cognitive or academic outcomes, but the evidence was predominantly cross-sectional and did not establish whether timing effects were independent of sleep duration (Dutil et al., 2022). Research on sleep consistency has also been characterized by varied definitions and metrics, including bedtime variability, wake-time variability, intraindividual variability, and social jetlag, with much of the work concentrated in adolescents (Nicholson et al., 2023). Reviews of later school start times provide useful evidence concerning a structural intervention, but their academic analyses have largely emphasized grades and standardized test scores rather than broader developmental pathways (Biller et al., 2022).

Taken together, the literature does not yet provide a clear comparative account of which sleep parameters have been studied in relation to which developmental outcomes, where findings are consistent, and where evidence remains limited to cross-sectional associations. Pooling all sleep measures and developmental outcomes into a single estimate risks conflating conceptually distinct exposures and outcomes. It may also obscure areas where the apparent volume of research is not matched by strong study designs or objective measurement. An evidence and gap map is well suited to this type of question because it applies a prespecified framework across multiple exposure and outcome domains, allowing identification of both concentrations of evidence and systematically under-researched areas (Snilstveit et al., 2016; White et al., 2020).

The present review therefore organized the evidence across four sleep domains: sleep duration, sleep quality or fragmentation, sleep timing or regularity, and daytime sleepiness. These exposures were cross classified against four outcome domains: executive function and self-regulation, attention and vigilance, emotional and behavioral regulation, and academic functioning. The outcome domains were selected to reflect processes relevant to learning and adaptive functioning during childhood and adolescence. Executive control and attention represent relatively proximal cognitive and regulatory capacities, whereas classroom behavior and academic performance are more distal outcomes shaped by family, school, socioeconomic, and other contextual influences. This framework was used to structure the synthesis and should not be interpreted as assuming that all included studies tested, or could establish, a single causal pathway from sleep to academic achievement.

Accordingly, this systematic review addressed the following question: among typically developing children and adolescents, how are objectively or subjectively measured sleep duration, sleep quality or fragmentation, sleep timing or regularity, and daytime sleepiness associated with executive function and self-regulation, attention and vigilance, emotional and behavioral regulation, and academic functioning? Beyond synthesizing the direction and consistency of reported associations, the review aimed to map the distribution and methodological strength of the evidence across sleep–outcome combinations. Particular attention was given to study design and sleep measurement approach to identify domains supported by longitudinal or experimental evidence and those in which conclusions continue to rely primarily on cross-sectional or self-reported data.

## 2. Materials and Methods

### 2.1. Protocol Registration and Reporting Standards

This systematic review and evidence and gap map were conducted in accordance with the Preferred Reporting Items for Systematic Reviews and Meta-Analyses (PRISMA) 2020 statement (Page et al., 2021). The review protocol was registered prospectively on the International Prospective Register of Systematic Reviews (PROSPERO; ID: CRD420261438604).

### 2.2. Eligibility Criteria

Eligibility criteria were defined a priori using the Population–Exposure–Comparator–Outcome (PECO) framework above and were applied consistently at both the title/abstract and full-text screening stages.

Population. Typically developing children and adolescents aged 6 to 19 years, including general population samples, school-based cohorts, and community samples. Studies conducted exclusively in clinical populations (obstructive sleep apnea, epilepsy, attention-deficit/hyperactivity disorder) were excluded unless normative or non-clinical subgroup data were reported separately and could be extracted independently of the clinical sample.

Exposure. Objectively or subjectively measured sleep parameters, including sleep duration, sleep regularity/timing, sleep fragmentation, sleep efficiency, and sleep quality, assessed via actigraphy, polysomnography (PSG), sleep diaries, or validated questionnaires (the Children’s Sleep Habits Questionnaire [CSHQ], the Pittsburgh Sleep Quality Index [PSQI] or pediatric adaptations thereof). Daytime napping was not separately operationalized as an exposure category in this review. Midday or scheduled napping constitutes a distinct sleep-opportunity intervention rather than a nighttime sleep parameter, and napping-specific search terms (e.g., ‘nap,’ ‘napping’) were not included in the database search strings (Table 1); consequently, napping-focused studies were not systematically retrieved by this review’s search strategy rather than being excluded at the screening stage.

Comparator. Not restricted a priori. Eligible comparators included naturalistic associations between sleep parameters and outcomes (shorter vs. longer habitual sleep duration) as well as experimental or quasi-experimental manipulations of sleep duration or timing (sleep restriction/extension protocols, delayed school start times).

Outcome. Cognitive and behavioral outcomes, operationalized as attention, executive function, self-regulation, academic performance, or behavioral problems (including hyperactivity, impulsivity, and emotion/conduct difficulties), assessed using validated neuropsychological tasks, standardized informant- or self-report rating scales, or objective academic indices (grade point average).

Study design. Cross-sectional, longitudinal/prospective, and experimental designs (including randomized controlled trials, crossover trials, and within-subject sleep-manipulation protocols) were eligible. Systematic reviews and meta-analyses were eligible for background contextualization only and were excluded from primary data extraction and synthesis.

Additional inclusion criteria: (1) peer-reviewed journal publication; (2) human participants; (3) English-language full text.

Exclusion criteria: (1) studies conducted exclusively in clinical or medical populations without data applicable to typically developing children; (2) studies restricted to participants younger than 6 or older than 19 years without age-stratified data permitting extraction of the eligible age range; (3) narrative reviews, commentaries, editorials, or opinion papers without primary data; (4) studies reporting only physiological sleep correlates without a behavioral or cognitive outcome measure; (5) non-English-language full text; (6) conference abstracts, theses, or other sources for which full peer-reviewed text could not be obtained.

### 2.3. Information Sources and Search Strategy

A systematic search was conducted across four bibliographic databases: PubMed, PsycINFO, Scopus, and Web of Science Core Collection, from database inception to May 2026, with no lower date limit applied. Search strategies combined controlled vocabulary (MeSH terms) and free-text keywords across three concept blocks: (a) sleep parameters and measurement (sleep duration, sleep quality, sleep fragmentation, actigraphy, polysomnography); (b) the pediatric/adolescent population (child, adolescent, school-age); and (c) cognitive and behavioral outcome constructs (executive function, self-regulation, inhibitory control, hyperactivity, academic performance). Strategies were independently adapted to the syntax and indexing conventions of each database and are reported in full in Table 1. Searches were restricted to English-language, peer-reviewed journal articles; reviews, meta-analyses, editorials, letters, and comments were excluded at the database-filter level where functionality permitted, with residual non-primary records removed during screening. No supplementary search methods, such as forward or backward citation chasing, reference-list hand-searching, or direct author contact, were undertaken. The search was designed to capture the broader literature on sleep characteristics and cognitive and behavioral functioning in children and adolescents rather than to maximize sensitivity independently for each exposure–outcome cell subsequently represented in the EGM, which was applied during evidence classification and synthesis. This represents an important methodological limitation because restricting retrieval to four databases without supplementary searching, together with uneven representation of individual constructs in the search terminology, may have increased the likelihood of under-retrieval in less densely studied domains, particularly sleep timing/regularity, daytime sleepiness, attention/vigilance, and academic functioning. Accordingly, sparsely populated or empty EGM cells should be interpreted as reflecting the evidence identified through the present search strategy rather than as confirmation that no relevant primary research exists.

### 2.4. Study Selection

Two reviewers (I.K. and M.T.) independently performed the search based on the developed search strategy and the title screening against the predefined eligibility criteria. Discrepancies were resolved through discussion and consensus with the third reviewer (K.S.). All records retrieved from the four databases were exported into Rayyan web-based platform, and duplicate records were identified and removed prior to screening. Study selection proceeded in two sequential stages, consistent with PRISMA 2020 guidance.

In the first stage, titles and abstracts of all unique records were screened independently by two reviewers (I.K. and M.T.) against the predefined eligibility criteria. Records that clearly did not meet inclusion criteria—including those involving exclusively clinical populations, participants outside the 6–19-year age range, or outcomes unrelated to the four defined cognitive/behavioral domains—were excluded at this stage.

In the second stage, full-text reports of all records retained after title/abstract screening were retrieved and independently assessed for eligibility by the same two reviewers. Disagreements at either screening stage were resolved through discussion and consensus; where consensus could not be reached, a third reviewer adjudicated (K.S.). Reasons for full-text exclusion were recorded for every excluded report. The principal reasons for exclusion were: participant population outside the normotypic developmental scope of the review (exclusively clinical or neurodevelopmental samples); absence of an outcome measure within the four defined cognitive/behavioral domains; and non-original study design (narrative reviews, commentaries).

Full documentation of exclusion reasons is provided in Supplementary Table S1.

### 2.5. Data Extraction

Data were extracted independently by two reviewers (I.K. and M.T.) using a structured, piloted data-extraction form; discrepancies were resolved through discussion and consensus with the third reviewer (K.S.). For each included study, the following data were extracted: (1) bibliographic characteristics (first author, publication year, journal, country); (2) study characteristics (design, sample size, age range or developmental stage, population description); (3) sleep exposure, including its operational definition and measurement method (actigraphy, polysomnography, sleep diary, or validated questionnaire); (4) outcome domain, classified into one of four a priori categories; (5) outcome measure and instrument (Continuous Performance Test, Behavior Rating Inventory of Executive Function [BRIEF], Conners’ Rating Scales, grade point average); (6) quantitative findings, including effect sizes (Cohen’s d, correlation coefficients, odds ratios) and 95% confidence intervals where reported, and the direction of association; (7) covariates adjusted for in analysis; (8) reported effect moderators, where available; and (9) indicators of methodological quality relevant to risk-of-bias appraisal. When a study reported multiple effect sizes for the same sleep–outcome association (across subscales of a single instrument), the primary or composite estimate most directly aligned with the defined outcome domain was prioritized for synthesis, and this decision rule was applied consistently across studies.

### 2.6. Outcomes and Exposure Domains for Evidence Synthesis

Because included studies were heterogeneous in sleep measurement, outcome instrumentation, and effect-size metrics, sleep exposures and outcomes were classified into a priori analytic domains to structure synthesis, consistent with the theoretical framework.

Sleep exposures were grouped into four domains: (a) sleep duration or experimental sleep restriction/extension; (b) sleep quality, fragmentation, or efficiency; (c) sleep timing, regularity, variability, chronotype, or social jetlag; and (d) daytime sleepiness. Outcomes were grouped into four developmental domains: (a) executive function and self-regulation; (b) attention and vigilance; (c) emotion or behavior regulation; and (d) academic functioning. A single study could contribute data to more than one outcome domain when multiple relevant outcomes were reported, and study–outcome combinations (rather than studies alone) constituted the unit of synthesis for the evidence and gap map.

### 2.7. Risk of Bias and Methodological Quality Assessment

Methodological quality of included studies was appraised independently by two reviewers using the National Institutes of Health (NIH) National Heart, Lung, and Blood Institute (NHLBI) Quality Assessment Tools, with the design-appropriate instrument applied to each study: the Quality Assessment Tool for Observational Cohort and Cross-Sectional Studies was applied to cross-sectional and longitudinal/prospective designs, and the Quality Assessment Tool for Controlled Intervention Studies was applied to randomized, crossover, and within-subject experimental sleep-manipulation designs. Each domain was rated and an overall quality rating of good, fair, or poor was assigned per study, following NIH NHLBI scoring guidance. Disagreements between reviewers were resolved through discussion and consensus, with third-reviewer adjudication where required.

### 2.8. Data Synthesis: Evidence and Gap Map Approach

Given substantial heterogeneity across included studies in study design, sleep measurement, outcome instrumentation, and effect-size reporting, a conventional pooled meta-analysis was not considered an appropriate or valid primary synthesis method. The review therefore employed a theory-driven evidence and gap map (EGM) approach (Snilstveit et al., 2016; White et al., 2020) to characterize the distribution, consistency, and methodological strength of available evidence across the predefined sleep-exposure and outcome domains.

The evidence and gap map was constructed by cross-tabulating the four sleep-exposure domains against the four outcome domains, generating sixteen evidence cells. For each study–outcome link contributing to a cell, the following characteristics were coded: (1) direction of association, classified as favoring adequate/longer/higher-quality sleep, mixed, null, or unclear; (2) study design, classified as experimental/quasi-experimental, longitudinal, cross-sectional, or other/unclear; (3) sleep-measurement quality, classified as higher (objective measures: actigraphy or polysomnography), moderate (sleep diaries or validated questionnaires), or unclear (insufficiently described measurement); and (4) covariate adjustment, classified as no/unclear, basic, moderate, or high, according to the extent to which major developmental and sociodemographic confounders (e.g., age, sex, socioeconomic status) were accounted for in the analytic model.

Cell-level evidence strength was rated using a four-tier classification, applied a priori: stronger evidence required (i) contributions from multiple studies, (ii) at least one experimental or quasi-experimental study, (iii) higher-quality (objective) sleep measurement in at least two contributing study-links, and (iv) a dominant direction of association favoring adequate sleep; moderate evidence required at least two contributing studies and either an experimental/quasi-experimental or a longitudinal design contribution; preliminary evidence was assigned to any populated cell that did not meet the criteria for moderate or stronger evidence, including cells supported by a single study or by multiple studies restricted to cross-sectional designs; and evidence gap was assigned to cells with no contributing studies. The evidence-strength map translated cell-level study counts and design/measurement profiles into the four-tier strength classification described above.

This synthesis was supplemented by a narrative summary organized by sleep-exposure domain, describing the direction, consistency, and methodological characteristics of evidence within each domain, including divergent or null findings where present. Analysis and visualization were conducted in R, version 4.5.1 (R Foundation for Statistical Computing, Vienna, Austria) (R Core Team, 2025).

We elected to develop a bespoke, EGM-specific four-tier classification rather than applying GRADE, because GRADE is designed to rate confidence in a specific pooled effect estimate for a given outcome, and its default assumptions are not well suited to a review that explicitly forwent meta-analysis due to cross-study heterogeneity in exposure and outcome measurement. Our four-tier scheme instead evaluates the volume, design mix, and measurement objectivity of evidence within each of the sixteen predefined sleep-exposure by outcome-domain cells, consistent with established EGM methodology (Snilstveit et al., 2016; White et al., 2020), which does not itself mandate GRADE-based cell rating. We nonetheless recognize this as a deliberate departure from a widely used external framework and discuss its interpretive limitations, including its tendency to reward design diversity over design strength, in Section 4.1.

## 3. Results

### 3.1. Study Selection Results

The database search identified a total of 10,437 records: PubMed (MEDLINE) (*n* = 3240), Scopus (*n* = 5211), Web of Science (*n* = 198), and PsycINFO (*n* = 1788). After removal of 4150 duplicate records, 6287 unique records remained for title and abstract screening. No records were excluded by automation tools or for other pre-screening reasons. Title and abstract screening resulted in the exclusion of 5644 records that did not meet the predefined eligibility criteria, leaving 643 reports sought for full-text retrieval; all 643 were successfully retrieved (*n* = 0 not retrieved).

**Figure 1:**
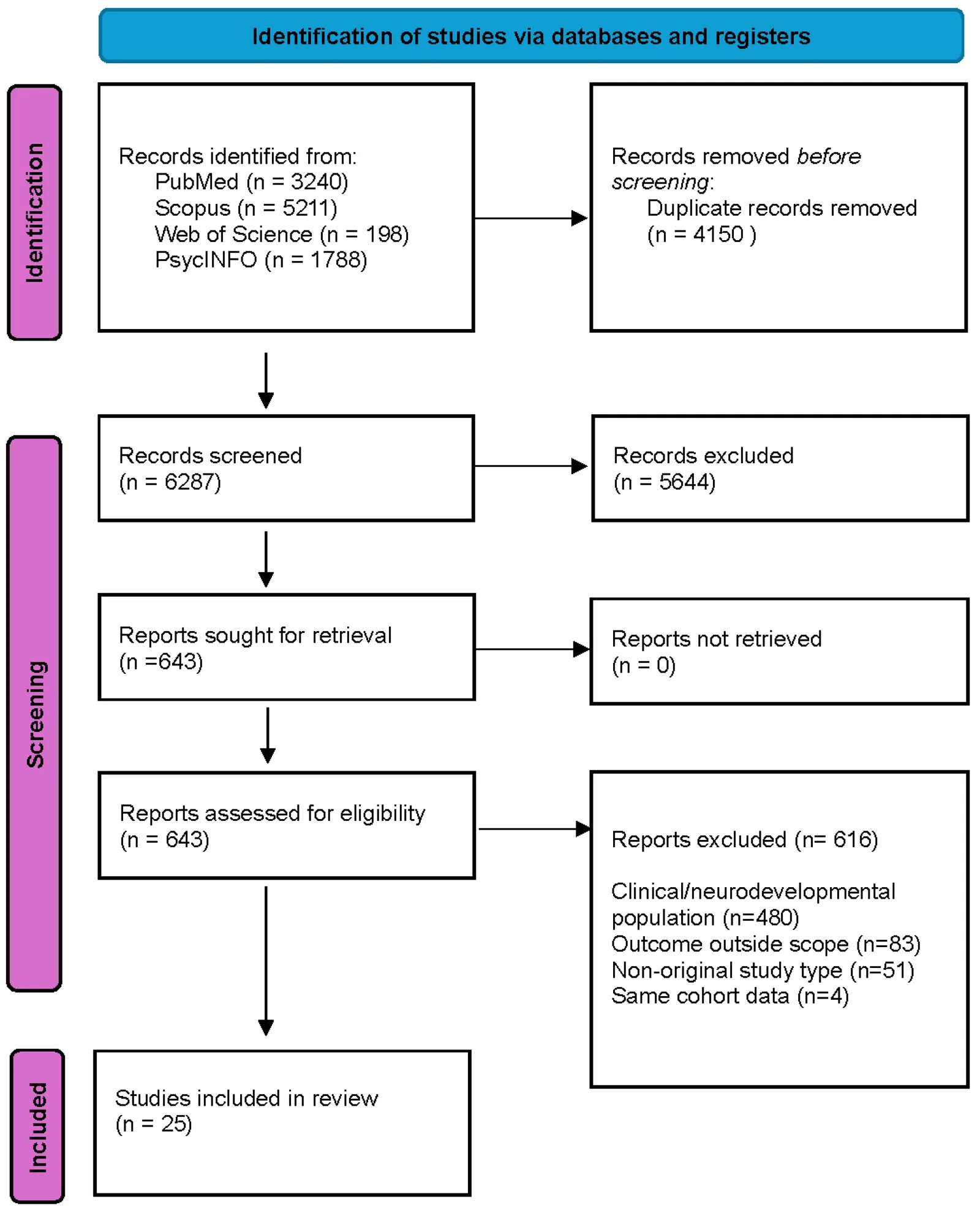
PRISMA 2020 flow diagram illustrating the study selection process.

Full-text assessment of these 643 reports resulted in the exclusion of 618 reports for the following reasons: restriction of the study population to clinical or neurodevelopmental samples outside the normotypic developmental scope of this review (*n* = 480); absence of an outcome measure within the four predefined cognitive or behavioral domains (*n* = 83); and non-original study design, encompassing narrative reviews, commentaries, and editorials (*n* = 51). A further four reports were excluded on the grounds that they drew on cohorts already represented by a more comprehensive included study (El-Sheikh et al., 2019; Paavonen et al., 2009; Sadeh et al., 2002; Yang et al., 2022); in each case, the report providing the fuller data was retained. A total of 25 studies met all eligibility criteria and were included in the review. The full identification, screening, and inclusion process is summarized in the PRISMA 2020 flow diagram (Figure 1).

### 3.2. Characteristics of Included Studies

The 25 studies were published between 2003 and 2026 and included cross-sectional, longitudinal/prospective, and experimental or quasi-experimental designs. Study populations comprised typically developing children and adolescents, with sample sizes ranging from small experimental studies to large population-based cohorts. Sleep was assessed using objective measures, including actigraphy and polysomnography, as well as subjective measures such as sleep diaries and validated sleep questionnaires. The most frequently examined sleep domains were sleep duration, sleep quality or fragmentation, sleep timing/regularity, and daytime sleepiness. Outcomes were grouped into executive function/self-regulation, attention/vigilance, emotion or behavior regulation, and academic functioning. Overall, the included studies provided heterogeneous but broadly complementary evidence linking poorer, shorter, more irregular, or sleepier sleep profiles with less favorable cognitive, behavioral, emotional, or academic outcomes. A full description of the study characteristics, sleep measures, outcome instruments, main findings, and methodological quality ratings is presented in Table 2.

### 3.3. Evidence and Gap Map Analysis

**Figure 2:**
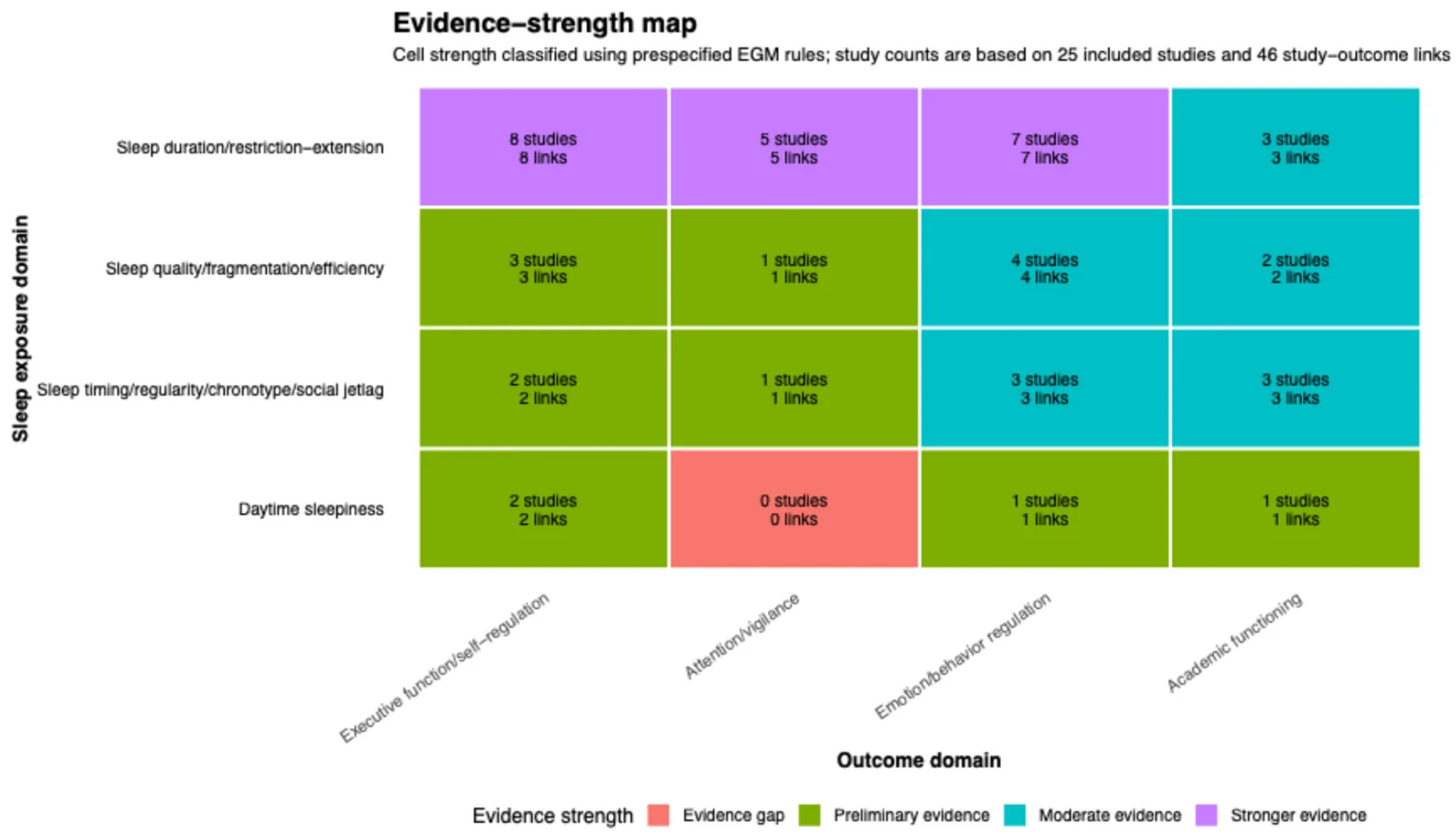
Evidence-strength map showing cell-level strength based on predefined EGM criteria. Each cell reports the number of contributing studies and the number of study–outcome links coded within that sleep-exposure-by-outcome-domain combination; cell shading denotes the four-tier evidence-strength classification (stronger, moderate, preliminary, evidence gap). A study may contribute more than one link within a cell. “Evidence gap” denotes the absence of contributing studies among records identified by the present search and should not be interpreted as proof that no relevant studies exist.

Because included studies varied substantially in design, sleep-measurement modality, outcome instrumentation, and effect-size reporting, findings were synthesized using a theory-driven EGM rather than pooled meta-analysis. Across the 25 included studies, 46 study–outcome links were coded across four predefined sleep-exposure domains and four outcome domains. Figure 2 summarizes the distribution and cell-level strength of evidence across these domains. Stronger evidence was concentrated in the sleep duration/restriction–extension domain for executive function/self-regulation, attention/vigilance, and emotion/behavior regulation, reflecting the greater number of contributing studies and the presence of longitudinal and experimental or quasi-experimental evidence. Moderate evidence was identified for sleep duration in relation to academic functioning, for sleep quality/fragmentation/efficiency in relation to emotion/behavior regulation, and for sleep timing/regularity/chronotype/social jetlag in relation to both emotion/behavior regulation and academic functioning. The remaining populated cells were classified as preliminary evidence. No eligible study linking daytime sleepiness with attention/vigilance was identified among the studies retrieved by the present search. Accordingly, this cell was classified as an evidence gap within the retrieved evidence base. Given the breadth of the original search terminology, however, this classification should not be interpreted as confirming the absence of relevant research outside the retrieved literature. Overall, the map demonstrates that the evidence base is most developed for sleep duration/restriction–extension and considerably less developed for daytime sleepiness and several sleep quality and sleep timing outcome combinations.

**Figure 3:**
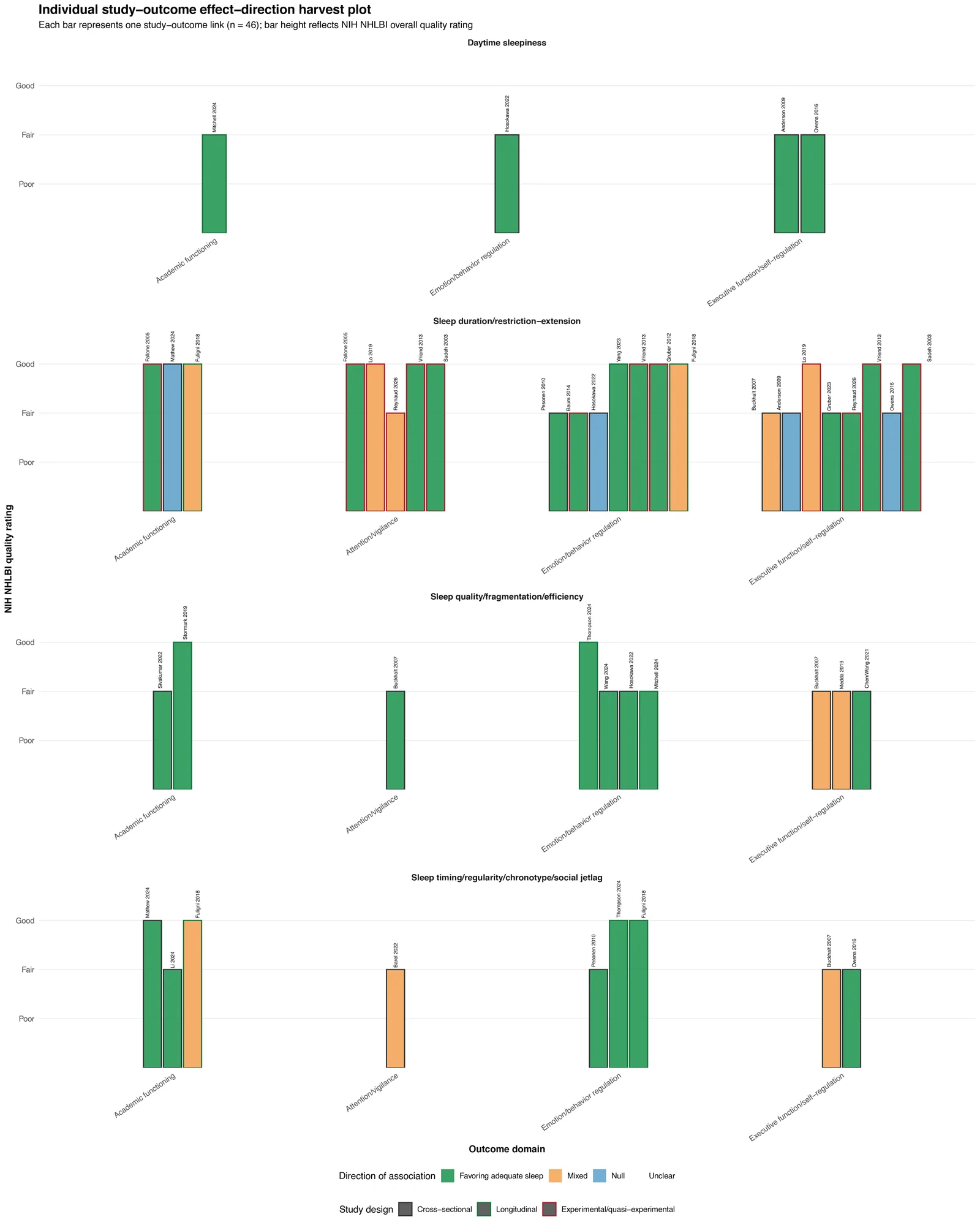
Individual study–outcome effect-direction harvest plot.

Figure 3 presents the individual study–outcome links contributing to the evidence and gap map. Across the 46 extracted links, the dominant pattern favored adequate sleep, defined as longer or experimentally extended sleep duration, better sleep quality or efficiency, lower fragmentation, more regular or developmentally aligned sleep timing, and lower daytime sleepiness. This pattern was visible across all four developmental outcome domains, although the density and methodological strength of evidence varied by sleep domain.

The largest and most methodologically diverse group of links came from the sleep duration/restriction–extension domain. This domain included cross-sectional, longitudinal, and experimental or quasi-experimental evidence and contributed links across executive function/self-regulation, attention/vigilance, emotion/behavior regulation, and academic functioning. Most links in this domain favored adequate sleep, particularly for executive function/self-regulation and emotion/behavior regulation. However, several mixed or null findings were also observed, indicating that the association between sleep duration and developmental outcomes was not uniform across all outcomes, measures, and study designs.

For sleep quality/fragmentation/efficiency, favorable directions of association predominated for emotion/behavior regulation and academic functioning. All four links involving emotion/behavior regulation and both links involving academic functioning favored adequate sleep, as did the single attention/vigilance link. In contrast, the three links involving executive function/self-regulation showed heterogeneous effect directions, with two classified as mixed and one favoring adequate sleep. This directional heterogeneity is distinct from the cell-level evidence-strength classification in Figure 2, where the sleep quality × executive function/self-regulation cell was classified as preliminary evidence according to the prespecified EGM criteria.

Overall, the harvest plot complements the evidence-strength map by showing the direction of individual study–outcome links. Favorable associations predominated, but mixed and null findings were also present, particularly within some sleep duration and sleep quality cells. Together, the two figures demonstrate that the evidence base is uneven: sleep duration/restriction–extension is supported by the greatest number and methodological diversity of studies, whereas daytime sleepiness, sleep timing/regularity, and several sleep-quality outcome combinations remain comparatively underdeveloped.

**Figure 4:**
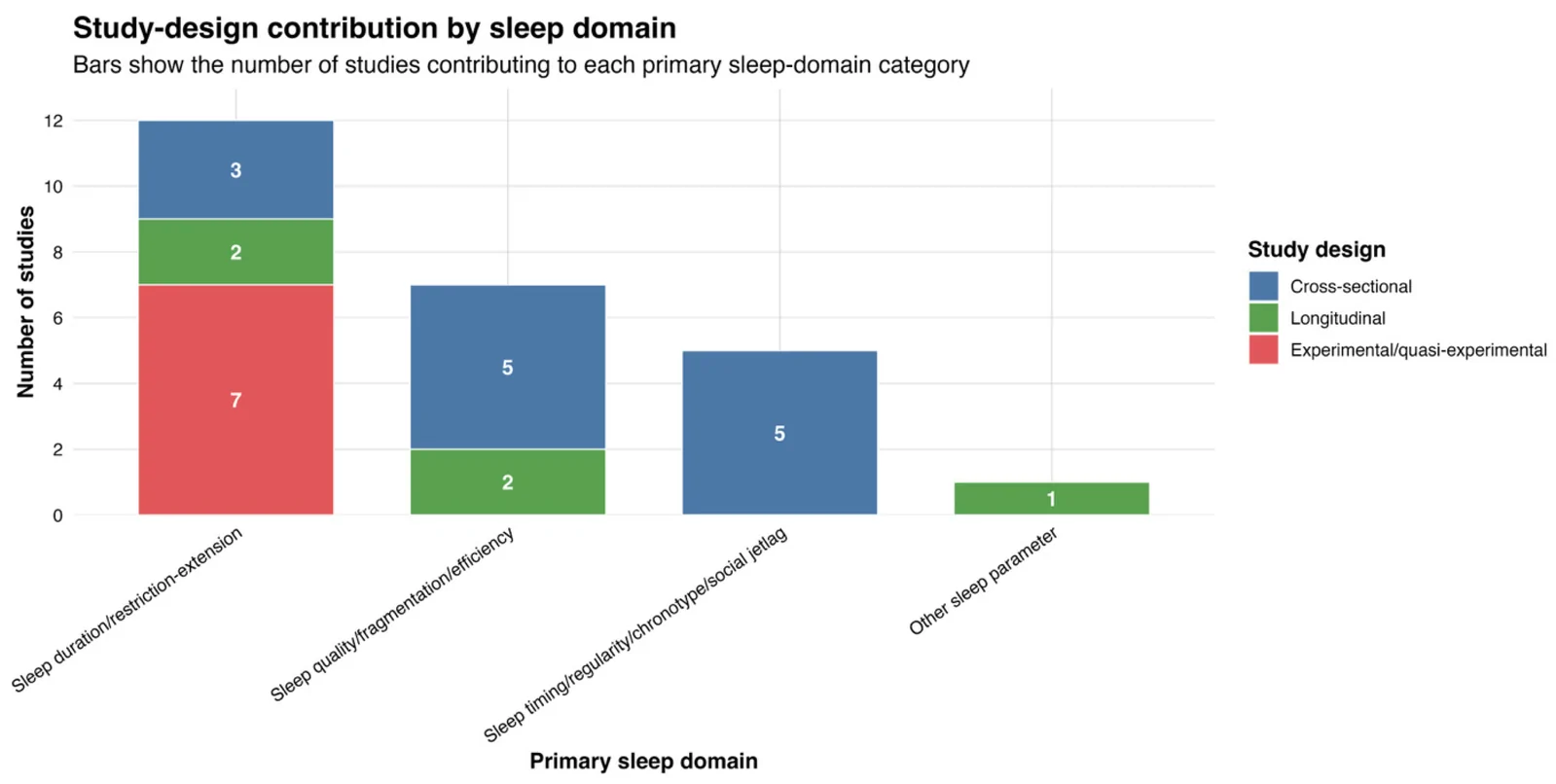
Study-design contribution by sleep domain.

Figure 4 shows the distribution of study designs across the primary sleep-domain categories. The largest body of evidence focused on sleep duration/restriction–extension, which accounted for 12 studies. This domain also had the greatest methodological diversity, including seven experimental or quasi-experimental studies, two longitudinal studies, and three cross-sectional studies. This indicates that sleep duration and sleep manipulation were the most developed areas of the evidence base and provided the strongest opportunity to evaluate temporality and potential causal effects.

Evidence for sleep quality/fragmentation/efficiency was more limited, with seven studies in total, including five cross-sectional and two longitudinal studies. No experimental or quasi-experimental studies primarily examined this domain. Sleep timing/regularity/chronotype/social jetlag was represented by five studies, all of which were cross-sectional, indicating that this domain remains largely observational. One longitudinal study was classified as other sleep parameter, reflecting a sleep-problem construct that did not map directly onto the main predefined domains.

**Figure 5:**
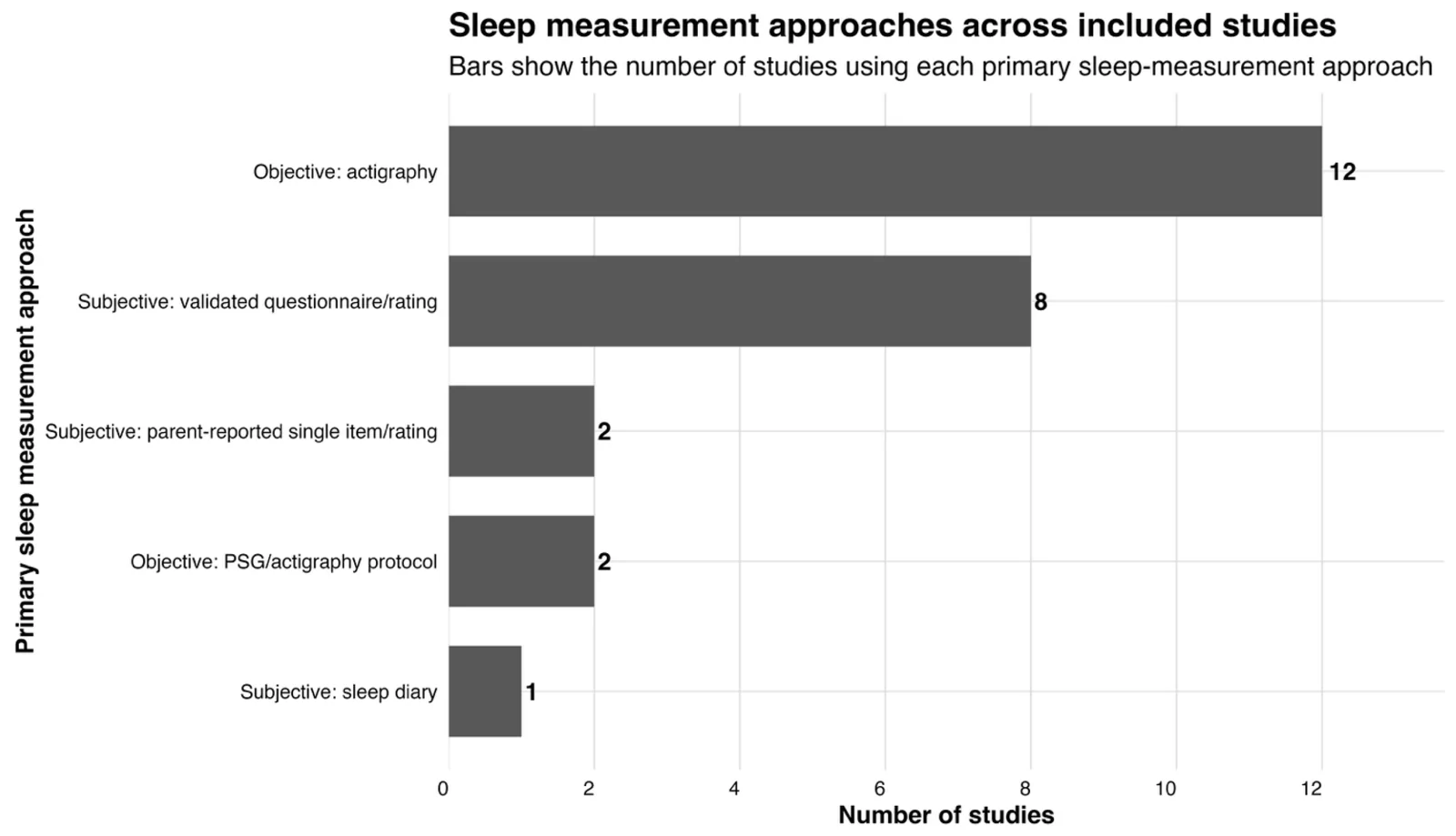
Sleep measurement approaches across included studies.

Figure 5 summarizes the primary sleep-measurement approaches used across the 25 included studies. Objective sleep assessment was common, with actigraphy used as the primary measurement approach in 12 studies, making it the most frequently used method. An additional two studies used a combined PSG/actigraphy protocol, further strengthening the objective measurement component of the evidence base.

Subjective sleep assessment was also frequently used. Validated questionnaires or rating scales were the primary sleep-measurement approach in eight studies, while parent-reported single-item or rating-based sleep measures were used in two studies. One study used a sleep diary as the primary sleep-measurement approach. Overall, the figure shows that nearly half of the included studies relied primarily on actigraphy, but a substantial proportion used subjective measures. This indicates that the evidence base includes a meaningful objective sleep-measurement component, although several sleep domains, particularly broader sleep-problem constructs, sleepiness, chronotype, and social jetlag, remain dependent on questionnaire, diary, or parent-report assessments.

### 3.4. Methodological Quality of Included Studies

**Figure 6:**
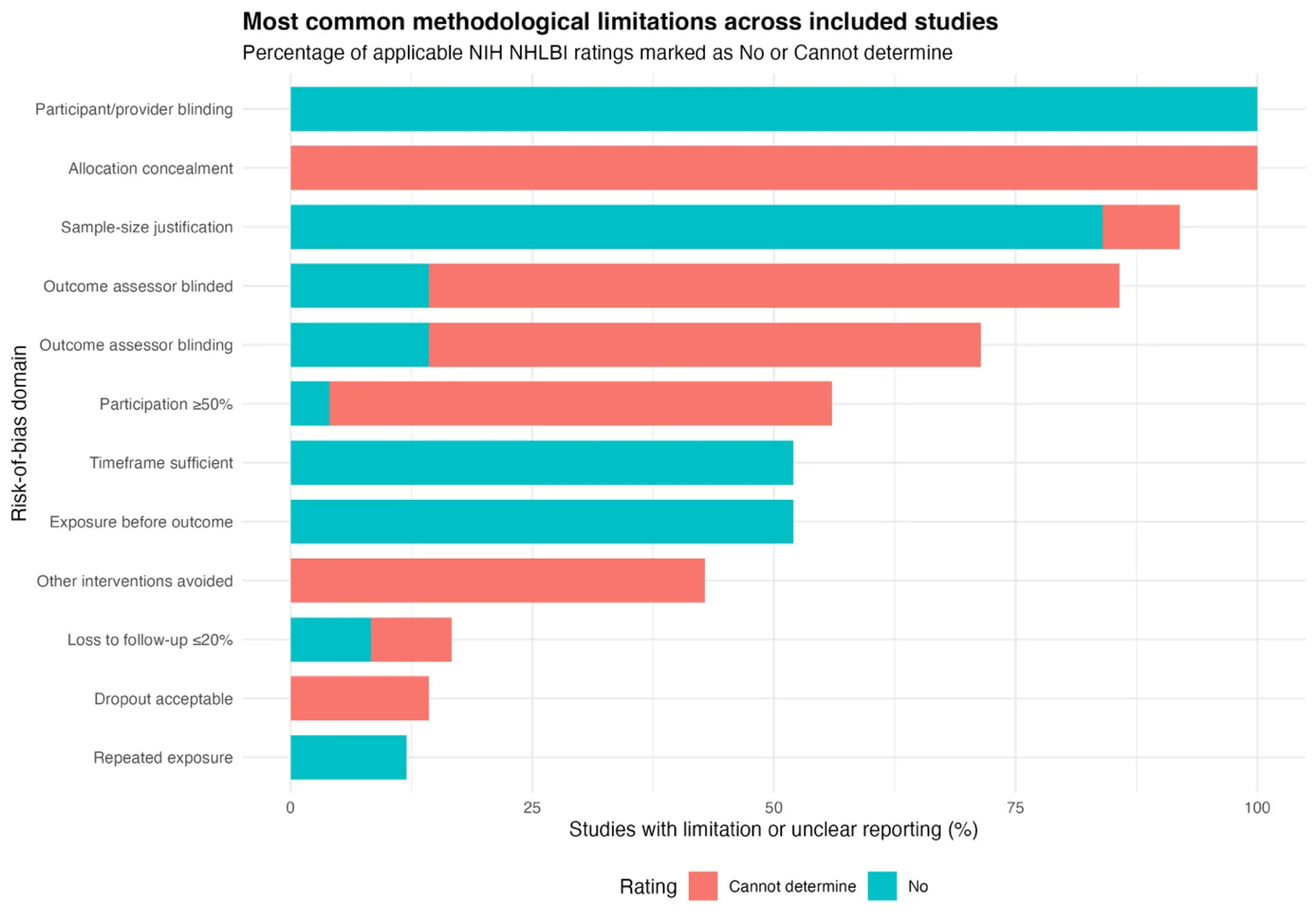
Most common methodological limitations across included studies.

Methodological quality was assessed using the design-appropriate NIH NHLBI quality appraisal tools. Overall, 10 studies were rated as good quality, 15 as fair quality, and none were rated as poor quality. The item-level methodological appraisal results are presented in Supplemental Figure S1, while the most frequent methodological limitations are summarized in Figure 6. The most common limitations were related to features that are difficult to address in sleep research, particularly lack of participant or provider blinding in intervention studies and unclear or absent allocation concealment. Across the full evidence base, the most frequent observational limitations were absence of sample-size justification, unclear participation rates, limited ability to establish temporality in cross-sectional studies, and incomplete or unclear reporting of blinded outcome assessment. Several observational studies also had limited capacity to control residual confounding, particularly when sleep and behavioral outcomes were both assessed using subjective questionnaires. Among longitudinal studies, attrition and follow-up reporting were additional concerns, although the larger longitudinal cohorts generally used stronger adjustment strategies.

For experimental and quasi-experimental studies, the main concerns were small sample sizes, incomplete blinding, and uncertainty around allocation concealment in some studies. However, several experimental studies strengthened internal validity through objective sleep monitoring, within-subject or randomized designs, and masked teacher or task-based outcome assessment where feasible. Overall, the risk-of-bias assessment indicated that the included literature was methodologically acceptable but not uniformly low risk.

## 4. Discussion

This evidence and gap map of 25 studies (46 study–outcome links) shows that shorter, poorer-quality, more irregular, or sleepier sleep profiles are consistently associated with less favorable executive, attentional, emotional, and academic outcomes in typically developing 6–19-year-olds, with the strongest and most methodologically diverse evidence for sleep duration and the weakest for daytime sleepiness. Rather than adding another isolated effect-size estimate, this map’s contribution is to show where the pediatric sleep-cognition literature is empirically load-bearing and where it still rests on thin, largely observational ground.

### 4.1. Interpreting the Pattern in Light of Current Evidence

Before turning to domain-specific patterns, it is important to state plainly what the uneven distribution of evidence does not imply. Sleep duration’s evidentiary predominance in this map reflects its comparative tractability as an experimentally manipulable, unidimensional parameter, more than it demonstrates that duration is developmentally more consequential than sleep quality, regularity, timing, or daytime sleepiness. The present findings are best read as identifying where the evidence base is currently strong enough to support causal inference, not as a ranking of which sleep dimension matters most for development. The concentration of strong evidence in the sleep duration/restriction–extension domain, and specifically its disproportionate contribution of experimental and quasi-experimental designs, is consistent with the broader literature showing that duration is both the most measurable and the most manipulable sleep parameter in pediatric research. This aligns with the largest existing meta-analysis in school-age children, which found a small but reliable positive association between sleep duration and cognitive performance, driven specifically by executive functioning and school performance rather than by attention or memory (Astill et al., 2012). The present map extends this picture across adolescence and across quasi-experimental designs, and its finding that duration effects were most robust for executive function and emotion/behavior regulation, but more mixed for attention, mirrors the domain-specificity Astill and colleagues first identified: sleep duration does not act as a uniform cognitive enhancer but preferentially supports the top-down regulatory processes subserved by prefrontal maturation, which is exactly the mechanistic pathway specified in the review’s guiding self-regulation framework. A recent longitudinal analysis of sleep health, executive function, and classroom behavior in 9-year-olds reinforces this directional and mechanistic reading, reporting that better sleep health predicted academic achievement in part through its association with executive function and classroom behavior rather than through a direct, unmediated pathway (Woods et al., 2024). Taken together, these findings are consistent with a potential pathway in which sleep supports self-regulatory capacity, which may in turn contribute to academic functioning.

It is worth briefly situating these pediatric effect sizes against the adult literature. Experimentally induced sleep loss produces larger acute cognitive decrements in young adults (Hedges’ g = −0.62) (Rajeev et al., 2025) than the small habitual-duration effects seen in children (Astill et al., 2012), and a lifespan meta-analysis similarly suggests adolescents are comparatively resilient to short-term sleep disruption relative to older adults (Xia et al., 2026). Children and adolescents therefore do not appear to show larger single-occasion cognitive costs of poor sleep than adults; if anything, the reverse holds. This reframes rather than undermines the case for prioritizing pediatric sleep: the argument is not that a given poor night costs a child more than an adult, but that childhood and adolescence coincide with an active, experience-dependent period of prefrontal synaptic pruning during which sleep-dependent consolidation processes are thought to support the formation of the executive-function circuitry these outcomes depend on (Baker et al., 2025; Selemon, 2013). Because this maturational window occurs only once, and because school-age sleep patterns are prospectively associated with later executive and academic trajectories (Woods et al., 2024), chronic pediatric sleep disruption plausibly carries cumulative, trajectory-shaping consequences that a physiologically mature adult brain does not face in the same way.

The comparatively weaker and more observational evidence found for sleep quality/fragmentation and sleep timing/regularity/chronotype also converges with the recent literature, and here the map’s gap analysis is arguably as informative as its positive findings. Large-cohort chronotype and social-jetlag studies using the Adolescent Brain Cognitive Development sample have shown that greater social jetlag and later chronotype predict poorer crystallized cognition and academic performance even after adjustment for core demographics, but these effects are cross-sectional and modest in size (Li et al., 2024). A related ABCD analysis incorporating socioeconomic status, latitude, and seasonality found that chronotype and social jetlag were negatively associated with vocabulary, reading, and working-memory scores, while explicitly cautioning that the cross-sectional design precluded causal inference (Burns et al., 2025). The present map’s classification of this entire domain as cross-sectional and, at best, moderate-strength evidence is therefore not a limitation unique to this review—it accurately reflects a field-wide absence of experimental circadian-timing manipulations in normative pediatric samples, in contrast to the much better-developed experimental base for sleep duration.

The mixed and occasionally null findings within the duration domain itself also deserve analytic attention rather than being treated as noise. This heterogeneity is consistent with evidence that different sleep-measurement modalities appear to capture different underlying constructs: studies contrasting actigraphy-derived duration with questionnaire-based sleep disturbance have found that habitual sleep problems, rather than duration per se, are what track most closely with parent- or teacher-rated executive function, suggesting that duration and continuity/quality are only weakly interchangeable proxies for “adequate sleep” (Chen et al., 2021). This measurement heterogeneity plausibly explains why the present map found duration effects to be strong for some outcome domains and mixed for others: the true exposure of developmental interest is arguably a multidimensional sleep-health construct, and duration-only operationalizations may underestimate the association wherever fragmentation or irregularity is the more proximal mechanism.

A further limitation of the duration literature synthesized here, and of the broader field, is that virtually none of the included studies operationalized individual physiological sleep need. Sleep duration was instead evaluated against absolute thresholds, age-based normative recommendations, or standardized restriction/extension protocols applied uniformly across participants, an approach that treats a given number of hours as equally adequate or inadequate for every child despite well-documented interindividual variability in biological sleep requirement. A child whose intrinsic need is seven hours and a child who requires nine hours may both be coded as sufficient or insufficient sleepers under the same absolute cutoff, which could attenuate true associations between adequate sleep and outcomes in ways not visible from the study-level data synthesized here.

A related complication for the ‘more adequate sleep is better’ framing comes from Fuligni et al. (2018), whose longitudinal data indicated that academic achievement peaked at a shorter sleep duration than the duration associated with optimal mental health—a curvilinear, outcome-specific pattern rather than a monotonic one (Fuligni et al., 2018). If replicated, this suggests a single duration target is unlikely to be simultaneously optimal for every developmental outcome domain, and that academic-functioning outcomes in particular may follow a different dose–response shape than emotional or behavioral outcomes. Because only one included study modeled sleep duration nonlinearly, we cannot determine how widespread this pattern is; we flag it as a hypothesis meriting explicit nonlinear modeling in future longitudinal work rather than as an established curvilinear relationship.

For emotion/behavior regulation, the map’s consistent directional signal is broadly compatible with the experimental sleep-restriction literature, though the underlying mechanism remains unsettled. Behavioral emotion-regulation deficits after acute sleep loss have historically been attributed to a prefrontal–amygdala ‘disconnect,’ but a controlled fMRI study found the expect ed behavioral impairment without a corresponding change in amygdala activity or connectivity (Tamm et al., 2019), and adolescent prefrontal–amygdala circuitry is itself still maturing (Selleck et al., 2018). The mechanism linking sleep loss to poorer youth emotion regulation therefore likely involves more than this connectivity account and remains mechanistically underspecified in the pediatric literature synthesized here.

Finally, the map’s clearest structural gap—the complete absence of study–outcome links between daytime sleepiness and attention/vigilance—is a genuine and somewhat counterintuitive finding, since sleepiness is conceptually the most attention-proximal sleep parameter. This gap likely reflects a methodological artifact of the field rather than a true absence of association: daytime sleepiness in pediatric research is disproportionately assessed through subjective, single-item, or parent-report instruments rather than objective vigilance protocols, which may have caused eligible studies to be coded into other exposure categories or excluded for insufficient outcome specificity. This is itself an evidence-base problem worth flagging analytically, not merely a screening artifact.

A related interpretive caveat concerns the evidence-strength scheme itself. Because ‘stronger’ evidence requires both an experimental/quasi-experimental design contribution and objective measurement in multiple links, the classification partly rewards design diversity and volume rather than design strength alone. A cell supported by several converging cross-sectional studies using objective sleep measures cannot reach ‘stronger’ status under this scheme, while a cell with a single modest experimental study plus objective cross-sectional evidence can. This dynamic is visible in the sleep quality/fragmentation × executive function/self-regulation cell (Section 3.3), which is supported by multiple studies but exclusively cross-sectional designs and therefore cannot reach a higher evidence tier under the prespecified classification (Rushworth et al., 2026). Because sleep duration is currently the only domain in which experimental manipulation is both ethically and logistically straightforward in pediatric samples, this scheme structurally favors the duration domain over sleep quality, timing, and regularity domains, independent of the intrinsic strength of the observational evidence in the latter (Byeon et al., 2025). We flag this as an interpretive caveat rather than revising the classification itself, since the a priori scheme was applied consistently across all sixteen cells; readers should weight cross-domain strength comparisons with this asymmetry in mind.

### 4.2. Practical Implications

These findings translate into a specific, evidence-graded set of implications rather than a generic “sleep matters” message. First, because the strongest and most causally interpretable evidence concerns sleep duration and its downstream effects on self-regulation and academic functioning, duration-focused, structurally enforced interventions are the most defensible near-term policy lever. Naturalistic and quasi-experimental evaluations of delayed school start times have shown consistent extensions in adolescent sleep duration and, in the largest recent difference-in-differences evaluation, measurable downstream gains, including fewer late arrivals and absences, a lower probability of behavioral referral, and higher GPA in policy-change schools relative to comparison schools (Alfonsi et al., 2020; Boergers et al., 2014). These findings are congruent with the present map’s conclusion that sleep duration is the domain best positioned to support causal, system-level intervention and support school start-time reform as a higher-yield target than individually delivered sleep-hygiene education. However, this implication is most directly applicable to adolescents and older school-aged children. For younger children, particularly those under 10 years of age, delaying school start times may not be the only, or necessarily the most feasible, structural response. Midday napping may represent an alternative or complementary strategy for increasing total sleep opportunity and supporting daytime functioning, yet this intervention was outside the scope of the present review and remains insufficiently represented in the evidence base (Lam et al., 2011; Liu et al., 2019). This constitutes an important gap for future research, especially in early primary-school settings where circadian needs, school schedules, and developmental sleep patterns differ from those of adolescents.

That prioritization is reinforced by the intervention literature: a systematic review of 21 randomized school-based sleep interventions involving nearly 11,000 students found that sleep-education programs, the most commonly tested intervention type, were generally ineffective at changing objectively or subjectively measured sleep behavior, whereas the single included study of a school start-time change produced a measurable increase in sleep duration. Practically, schools and health systems should therefore treat knowledge-based sleep education as, at best, a low-cost adjunct rather than a primary intervention and should direct policy efforts toward structural changes that increase sleep opportunity, including later start times for adolescents, reduced homework and extracurricular burden, limits on evening screen availability, and, for younger children, evaluation of developmentally appropriate midday nap opportunities.

Moreover, the moderate-to-preliminary strength of the timing/regularity and chronotype evidence implies that circadian-alignment strategies are conceptually promising but not yet ready for the same confidence of implementation as duration-focused policy. Given that social jetlag and late chronotype track independently with academic and cognitive outcomes even in very large cohorts (Burns et al., 2025; Li et al., 2024), schools serving predominantly adolescent (rather than younger) populations have a plausible, biologically motivated rationale for flexible or later scheduling, but clinicians and policymakers should present this as a promising, hypothesis-consistent direction rather than as an established causal lever, pending experimental confirmation.

Finally, because sleep-quality/fragmentation and continuity indices appear to carry incremental information beyond duration, particularly for emotion/behavior regulation and academic functioning, screening practices in pediatric and school-health settings that rely solely on parent-reported “hours of sleep” risk missing a clinically meaningful subset of children whose regulatory and academic difficulties are better explained by fragmented or poor-quality sleep than by short duration alone. Multidimensional sleep-health screening, even brief validated instruments, is therefore a low-cost, high-value addition to routine developmental and academic-support assessment.

## 5. Conclusions and Limitations

Taken together, this evidence and gap map substantiates its guiding premise: sleep is not a uniform, generic contributor to pediatric cognition but a multidimensional exposure whose components carry differentiated, mechanistically plausible, and unevenly evidenced relationships with self-regulation, attention, emotion regulation, and academic functioning. This multidimensional framing, rather than a duration-centric one, is the review’s central interpretive lens: the domains with less evidence here are not domains of lesser consequence, but domains awaiting the longitudinal and experimental designs that duration research has already accumulated. The map accomplishes its stated aim by showing precisely where this evidence base is strong enough to justify action—chiefly, sleep duration in relation to executive function, emotion/behavior regulation, and academic outcomes—and where confident causal claims remain premature, particularly for chronotype/timing and daytime sleepiness. This graded picture, rather than a flat “more sleep is better” conclusion, is what should guide the next generation of pediatric sleep policy and research: prioritizing structural, duration-extending interventions such as delayed school start times where the evidence is causally strongest, while treating circadian-alignment and sleep-quality strategies as scientifically promising but still evidentially provisional.

This review’s conclusions are bounded by the same evidentiary architecture it maps. The evidence and gap map approach was chosen because pooled meta-analysis was not viable given cross-study heterogeneity in sleep measurement, outcome instrumentation, and effect-size reporting, but this means that domain-level evidence-strength ratings summarize direction and design diversity rather than a quantitative pooled effect, and cells with few contributing links should not be read as reflecting a true absence of association. The restriction to four bibliographic databases without supplementary citation-chasing, reference-list hand-searching, or author contact is a substantive limitation of this review. Because this constraint was applied uniformly across exposure domains, it plausibly had an uneven practical impact: domains with a larger, more consistently indexed primary literature (sleep duration) were less vulnerable to under-retrieval than domains with a smaller, more dispersed literature (sleep timing/regularity, daytime sleepiness). Accordingly, the evidence-gap and preliminary-evidence classifications assigned to these sparser cells, particularly the complete gap identified for daytime sleepiness × attention/vigilance, should be read as reflecting the limits of a four-database search strategy as much as a genuine absence of primary research, and we caution readers and policymakers against treating these cells as confirmatory of a true evidence void. English-language and peer-reviewed-only inclusion criteria may have introduced language and publication bias. Because most contributing studies were cross-sectional or observational outside the duration domain, temporality and residual confounding remain genuine concerns for the timing/regularity and sleep-quality domains specifically, and the map’s evidence-strength classification, while transparent, still relies on the reviewers’ a priori tier definitions rather than an external validated scale. Because the search strategy did not include napping-specific terms, this review cannot speak comprehensively to daytime napping as a sleep-health intervention, an important omission given its potential relevance for younger children. Relatedly, because sleep adequacy was operationalized in the included literature via absolute duration, age norms, or standardized manipulation protocols rather than individually estimated sleep need, the map cannot distinguish whether null or mixed duration links reflect a true absence of association or a mismatch between nominal and physiologically required sleep. Although covariate adjustment was coded on a four-tier scale during data extraction and informed the evidence-strength classification indirectly via its role as a risk-of-bias proxy, we did not report the covariate-adjustment rating alongside the direction and design classification for each study–outcome link in the main text or supplementary files. Given our own concern about residual confounding in observational links, this is a transparency gap: readers cannot currently verify from the reported figures which specific study–outcome links were adequately adjusted for major confounders. We flag this as a priority for a future public update to this evidence and gap map. Finally, because study–outcome links rather than studies were the unit of synthesis, cell counts are not fully independent, and the map’s picture of “where evidence is strong” should be interpreted as a map of the literature’s current structure rather than a definitive statement about the underlying developmental biology.

Looking forward, we join calls for longitudinal and intervention designs that pair objective sleep assessment (actigraphy, polysomnography) with multidimensional sleep-health measurement and, where feasible, methods for estimating individual optimal sleep duration rather than relying solely on absolute or age-normed thresholds. Integrating such individualized estimates with experimental and longitudinal designs represents a plausible next step toward precision approaches to pediatric sleep health and would strengthen the evidence base underlying personalized sleep recommendations.

## Figures and Tables

**Table 1:** Search strategies used to identify studies on sleep parameters and cognitive and behavioral outcomes in school-age children and adolescents.

Database	Search Field(s)	Search String	Filters/Limits
PubMed	Title/Abstract + MeSH	(“Sleep”[Majr] OR “Sleep Wake Disorders”[Majr] OR “Circadian Rhythm”[Majr] OR “sleep duration”[tiab] OR “total sleep time”[tiab] OR “short sleep”[tiab] OR “insufficient sleep”[tiab] OR “sleep quality”[tiab] OR “sleep problems”[tiab] OR “sleep disturbances”[tiab] OR “sleep disturbance”[tiab] OR insomnia[tiab] OR bedtime[tiab] OR chronotype[tiab] OR “social jetlag”[tiab] OR “sleep regularity”[tiab] OR “sleep variability”[tiab] OR “sleep timing”[tiab] OR “sleep fragmentation”[tiab] OR “sleep efficiency”[tiab] OR actigraphy[tiab] OR polysomnography[tiab] OR “sleep diary”[tiab]) AND (“Child”[Mesh] OR “Adolescent”[Mesh] OR child[tiab] OR children[tiab] OR adolescent[tiab] OR adolescents[tiab] OR teenager[tiab] OR youth[tiab] OR “school-age”[tiab]) AND (“Executive Function”[Majr] OR “Self-Control”[Majr] OR “Attention Deficit Disorder with Hyperactivity”[Majr] OR “Impulsive Behavior”[Majr] OR “Aggression”[Majr] OR “Conduct Disorder”[Majr] OR self-regulation[tiab] OR “executive function”[tiab] OR “executive functions”[tiab] OR “inhibitory control”[tiab] OR “working memory”[tiab] OR “cognitive flexibility”[tiab] OR impulsivity[tiab] OR “emotion regulation”[tiab] OR externalizing[tiab] OR hyperactivity[tiab] OR “ADHD symptoms”[tiab] OR aggression[tiab] OR “conduct problems”[tiab] OR “behavior problems”[tiab] OR “behaviour problems”[tiab]) AND (“Journal Article”[pt] OR “Observational Study”[pt] OR “Clinical Trial”[pt]) NOT (animals[mh] NOT humans[mh]) NOT (“Review”[pt] OR “Systematic Review”[pt] OR “Meta-Analysis”[pt] OR “Editorial”[pt] OR “Letter”[pt] OR “Comment”[pt])	Humans; English
Scopus	TITLE-ABS-KEY	TITLE-ABS-KEY (“sleep duration” OR “total sleep time” OR “short sleep” OR “insufficient sleep” OR “sleep quality” OR “sleep problem” OR “sleep problems” OR “sleep disturbance” OR “sleep disturbances” OR insomnia OR bedtime OR chronotype OR “circadian preference” OR “social jetlag” OR “sleep regularity” OR “sleep variability” OR “sleep timing” OR “sleep fragmentation” OR WASO OR “sleep efficiency” OR actigraphy OR polysomnography OR “sleep diary”) AND TITLE-ABS-KEY(child OR children OR adolescent OR adolescents OR teenager OR teenagers OR youth OR “school-age” OR “school aged”) AND TITLE-ABS-KEY(“self-regulation” OR “effortful control” OR “executive function” OR “executive functions” OR “inhibitory control” OR “response inhibition” OR “working memory” OR “cognitive flexibility” OR impulsivity OR impulsive OR “impulse control” OR “emotion regulation” OR “emotional regulation” OR “emotion dysregulation” OR externalizing OR externalising OR hyperactivity OR “ADHD symptoms” OR aggression OR aggressive OR “conduct problems” OR “behavior problems” OR “behaviour problems”) AND NOT TITLE-ABS-KEY (animal OR animals OR mouse OR mice OR rat OR rats)	Document type: Article and short survey; Language: English
Web of Science	Topic (TI + TS)	TI = (sleep OR “sleep duration” OR “sleep quality” OR insomnia OR bedtime OR chronotype OR “social jetlag” OR “sleep regularity” OR “sleep timing” OR “sleep fragmentation” OR actigraphy OR polysomnography) AND TS = (child OR children OR adolescent OR adolescents OR teenager OR teenagers OR youth OR “school-age” OR “school aged”) AND TS = (“self-regulation” OR “effortful control” OR “executive function” OR “executive functions” OR “inhibitory control” OR “response inhibition” OR “working memory” OR “cognitive flexibility” OR impulsivity OR “impulse control” OR “emotion regulation” OR “emotional regulation” OR “emotion dysregulation” OR externalizing OR externalising OR hyperactivity OR “ADHD symptoms” OR aggression OR “conduct problems” OR “behavior problems” OR “behaviour problems”) NOT TS = (animal OR animals OR mouse OR mice OR rat OR rats)	Document type: Article and Early Access; Language: English
PsycINFO	Title/Abstract	(TI sleep OR TI “sleep duration” OR TI “sleep quality” OR TI insomnia OR TI bedtime OR TI chronotype OR TI “social jetlag” OR TI “sleep regularity” OR TI “sleep timing” OR TI “sleep fragmentation” OR TI actigraphy OR TI polysomnography) AND (child OR children OR adolescent OR adolescents OR teenager OR teenagers OR youth OR “school-age” OR “school aged”) AND (“self-regulation” OR “effortful control” OR “executive function” OR “executive functions” OR “inhibitory control” OR “response inhibition” OR “working memory” OR “cognitive flexibility” OR impulsivity OR “impulse control” OR “emotion regulation” OR “emotional regulation” OR “emotion dysregulation” OR externalizing OR externalising OR hyperactivity OR “ADHD symptoms” OR aggression OR “conduct problems” OR “behavior problems” OR “behaviour problems”)	Language: English; Document type: Academic Journals

Abbreviations: ADHD, attention-deficit/hyperactivity disorder; Majr, MeSH Major Topic heading (PubMed search tag restricting retrieval to records in which the term is a major focus of the article); MeSH, Medical Subject Headings; mh, MeSH Heading (PubMed search tag); pt, Publication Type (PubMed search tag); TI, Title (Web of Science and PsycINFO search field); tiab, Title/Abstract (PubMed search tag); TITLE-ABS-KEY, Title, Abstract, and Keywords (Scopus search field); TS, Topic (Web of Science search field encompassing title, abstract, author keywords, and Keywords Plus); and WASO, wake after sleep onset.

**Table 2:** Characteristics of the 25 included studies.

Study	Country	Design	Sample and Population	Sleep Exposure and Measurement	Outcome Domain(s) and Instruments	Main Finding	NIH Rating
(Sadeh et al., 2003)	Israel	Randomized experimental	n = 77; 9.1–12.2 years; regular 4th- and 6th-grade children	Sleep duration manipulated by 1 h restriction or extension for 3 nights; actigraphy and sleep logs	Executive function/self-regulation and attention; computerized neurobehavioral battery	Sleep restriction reduced alertness and impaired selected neurobehavioral functions; sleep extension showed the opposite pattern.	Good
(Fallone et al., 2005)	USA	Crossover experimental	n = 74; 6–12 years; healthy children screened for medical and psychological conditions	Sleep opportunity restriction across week-long schedules; actigraphy	Attention and academic functioning; masked teacher ratings of academic, attention, and behavior problems	Restricted sleep increased teacher-rated attention and academic problems, with limited effects on hyperactivity.	Good
(Buckhalt et al., 2007)	USA	Cross-sectional	n = 166; 7.2–11 years; African American and European American children across SES strata	Sleep duration, quality/fragmentation, and schedule variability; actigraphy, sleep logs, and child sleep report	Executive function/self-regulation and attention; Woodcock-Johnson III and reaction-time tasks	Sleep quality, duration, and schedule variability were associated with cognitive performance, with moderation by race and SES.	Fair
(Anderson et al., 2009)	USA	Cross-sectional	n = 236; 13–16 years; healthy community adolescents	Sleep duration and daytime sleepiness; modified Epworth Sleepiness Scale, 5–7 day actigraphy, and overnight PSG	Executive function/self-regulation; BRIEF Global Executive Composite and D-KEFS Tower Test	Daytime sleepiness, but not actigraphic sleep duration, was associated with poorer selected executive-function outcomes.	Fair
(Pesonen et al., 2010)	Finland	Cross-sectional	n = 280; ~8 years; Helsinki 1998 population cohort	Sleep duration and weekday–weekend regularity; 1-week actigraphy with sleep logs	Emotion/behavior regulation; mother- and father-rated Child Behavior Checklist scales	Shorter and more irregular sleep were associated with more behavioral and emotional problems.	Fair
(Gruber et al., 2012)	Canada	Randomized experimental	n = 34; 7–11 years; typically developing children without reported sleep, behavioral, medical, or academic problems	1 h sleep extension or restriction relative to baseline; actigraphy and diary	Emotion/behavior regulation; teacher-rated Conners Global Index and daytime sleepiness	Modest sleep extension improved teacher-rated emotional lability/restless-impulsive behavior; restriction worsened these outcomes.	Good
(Vriend et al., 2013)	Canada	Crossover experimental	n = 32; 8–12 years; typically developing children	Habitual, short-sleep, and long-sleep conditions using 1 h bedtime manipulation; actigraphy and diary	Executive function/self-regulation, attention, and emotion regulation; memory, attention, affect, and regulation tasks/scales	Short sleep impaired positive affect, emotion regulation, working memory, and aspects of attention compared with longer sleep.	Good
(Baum et al., 2014)	USA	Crossover experimental	n = 50; 14–17.9 years; healthy adolescents	Sleep restriction (6.5 h time-in-bed) versus healthy sleep (10 h time-in-bed) for 5 nights; actigraphy and diary	Emotion/behavior regulation; adolescent mood scales and parent/adolescent emotion-regulation reports	Experimental sleep restriction worsened mood and emotion regulation in adolescents.	Fair
(Owens et al., 2016)	USA	Cross-sectional	n = 2017; 12.1–18.9 years; public-school adolescents	School-night sleep duration, daytime sleepiness, and chronotype; self-report sleep survey and Morningness–Eveningness Scale	Executive function/self-regulation; BRIEF-2 Screening Self-Report	Greater sleepiness and eveningness were independently associated with lower self-regulation; sleep duration was not significant after adjustment.	Fair
(Fuligni et al., 2018)	USA	Longitudinal prospective	n = 421 at wave 1; ~15 years; Mexican American high-school adolescents with 1-year follow-up	Sleep duration and night-to-night variability; 14-night sleep diaries	Academic functioning and emotion/behavior regulation; achievement and internalizing/externalizing symptoms	Optimal sleep differed by outcome: more sleep was linked to better mental health, whereas academic achievement peaked at shorter durations; variability predicted more symptoms.	Good
(Lo et al., 2019)	Singapore	Parallel-group experimental	n = 58; 15–19 years; healthy adolescents in a dormitory protocol	Restricted sleep apportioned as split sleep versus continuous nocturnal sleep; PSG and actigraphy	Executive function/self-regulation and attention; PVT and cognitive battery	Under sleep restriction, split sleep preserved vigilance, working memory/executive function, processing speed, and mood better than continuous restricted sleep.	Good
(Medda et al., 2019)	Italy	Cross-sectional twin study	n = 382; 14–18 years; adolescent twins from the Italian Twin Registry	Sleep quality classified as good/non-good sleeper; Sleep Disorders Questionnaire	Executive function/self-regulation; CBCL/YSR-derived effortful control	Poorer self-reported sleep quality was associated with lower effortful control, but the association weakened in discordant-twin analyses.	Fair
(Stormark et al., 2019)	Norway	Longitudinal prospective	n = 3986; 7–13 years; Norwegian population-based child cohort	Difficulties initiating and maintaining sleep coded as concurrent, transitory, or persistent; parent report	Academic functioning; teacher-reported school performance, with SDQ mental-health covariates	Persistent, but not transitory, sleep problems predicted poor academic performance after adjustment for mental health.	Good
(Chen et al., 2021)	China	Cross-sectional	n = 204; 7.6–9.2 years; typically developing primary-school children	Habitual sleep disturbance; Chinese Sleep Self-Report	Executive function/self-regulation; Red-Blue Test, Wisconsin Card Sorting Test, and backward digit span	Self-reported sleep disturbance was associated with poorer inhibitory control and working memory and indirectly with cognitive flexibility.	Fair
(Barel & Tzischinsky, 2022)	Israel	Naturalistic repeated-measures	n = 104; 6–19 years; children and adolescents assessed across school and non-school days	Sleep duration, timing, and weekday/weekend patterns; 5–7 night actigraphy and sleepiness rating	Attention/vigilance; brief Psychomotor Vigilance Test	Sleep timing differences across school and non-school days were related to vigilant-attention performance, particularly among adolescents.	Fair
(Hosokawa et al., 2022)	Japan	Cross-sectional	n = 604; 12–14 years; first-year junior high-school students without developmental disability	Sleep quality, duration, efficiency, disturbances, and daytime arousal difficulty; parent-reported PSQI	Emotion/behavior regulation; parent-reported Strengths and Difficulties Questionnaire	Poor sleep quality, sleep difficulties, daytime arousal difficulties, and sleep disturbances were associated with higher behavioral problems.	Fair
(Sivakumar et al., 2022)	India	Cross-sectional	n = 791; 6–12 years; healthy schoolchildren in Chennai	Sleep habits and disturbances; parent-completed Children’s Sleep Habits Questionnaire	Academic functioning; school grades from two previous academic cycles	Altered sleep habits, particularly night waking/sleep disturbance, were associated with lower academic performance.	Fair
(Gruber et al., 2023)	Canada	Cross-sectional	n = 100; mean age 13.17 years; adolescents with and without subclinical insomnia symptoms	Objective sleep duration and insomnia symptoms; 6-night actigraphy and Insomnia Severity Index	Executive function/self-regulation; parent-rated BRIEF	Shorter objectively measured sleep duration, but not insomnia symptoms, predicted poorer daily executive functioning.	Fair
(Yang et al., 2023)	USA	Longitudinal prospective	n = 7884 baseline; 9–10 years; ABCD Study participants, with 5166 followed for neuroimaging	Parent-reported sleep duration categories from the Sleep Disturbance Scale for Children	Emotion/behavior regulation; UPPS-P impulsivity and corticostriatal connectivity	Shorter sleep duration was associated with higher affect-related impulsivity, partly mediated by corticostriatal connectivity.	Good
(Li et al., 2024)	USA	Cross-sectional	n = 6890; 11–14 years; ABCD Study year-2 adolescents	Chronotype and social jetlag; Munich Chronotype Questionnaire	Academic functioning and cognition; NIH Toolbox crystallized cognition and self-reported grades	Greater social jetlag/later chronotype predicted poorer crystallized cognition and academic performance with small effects.	Fair
(Mathew et al., 2024)	USA	Cross-sectional	n = 774–782; ~15 years; national diverse adolescent sample	Sleep duration, timing, efficiency, and variability; ~1 week actigraphy	Academic functioning; self-reported GPA, grades, school trouble, suspension/expulsion	Later sleep timing and greater variability, rather than average duration, were associated with poorer academic outcomes.	Good
(Mitchell et al., 2024)	USA	Longitudinal prospective	n = 278; 8–11 years; third- to fifth-grade students, 51.1% Hispanic/Latine	Sleep disturbance and sleep-related impairment profiles; PROMIS pediatric self-report forms	Emotion/behavior regulation and academic functioning; child- and teacher-reported symptoms, conduct, regulation, and academic performance	A profile combining elevated sleep disturbance and sleep-related impairment predicted worsening emotional, behavioral, and academic functioning over 6 months.	Fair
(Thompson et al., 2024)	USA	Longitudinal prospective	n = 199; ages 9, 10, 11, 17, and 18 years; Auburn University Sleep Study cohort	Sleep duration, efficiency, and variability; repeated 7-night actigraphy across five waves	Emotion/behavior regulation; parent-reported internalizing and externalizing symptoms	Better sleep efficiency predicted fewer internalizing/externalizing symptoms, whereas greater sleep variability predicted more symptoms across development.	Good
(Wang et al., 2024)	China	Cross-sectional mediation	n = 806; mean age 14.35 years; middle-school students	Subjective sleep quality and daytime dysfunction; selected PSQI components	Emotion/behavior regulation and self-control; DERS-16, social exclusion, and brief self-control scales	Poor sleep quality was linked to emotion-regulation difficulties through daytime dysfunction, social exclusion, and self-control pathways.	Fair
(Reynaud et al., 2026)	France	Cluster randomized school-start intervention	sleep n = 50; cognitive n = 73; 11.7–14.2 years; boarding-school early adolescents	One-hour delayed school start; actigraphy before and 6 months after randomization	Executive function/self-regulation and attention; Stroop task, SART, and sleepiness rating	Delaying school start by 1 h increased sleep duration and improved inhibitory control, with a trend toward better sustained attention.	Fair

Abbreviations: ABCD, Adolescent Brain Cognitive Development Study; BRIEF, Behavior Rating Inventory of Executive Function; BRIEF-2, Behavior Rating Inventory of Executive Function, Second Edition; CBCL, Child Behavior Checklist; DERS-16, Difficulties in Emotion Regulation Scale–Short Form; D-KEFS, Delis–Kaplan Executive Function System; EF, executive function; GPA, grade point average; NIH, National Institutes of Health; PSG, polysomnography; PROMIS, Patient-Reported Outcomes Measurement Information System; PSQI, Pittsburgh Sleep Quality Index; PVT, Psychomotor Vigilance Test; RCT, randomized controlled trial; SART, Sustained Attention to Response Task; SDQ, Strengths and Difficulties Questionnaire; SES, socioeconomic status; UPPS-P, Urgency, Premeditation, Perseverance, Sensation Seeking, Positive Urgency Impulsive Behavior Scale; and YSR, Youth Self-Report.

## Data Availability

The original contributions presented in this study are included in the article. Further inquiries can be directed to the corresponding author Indira Karibayeva (ik01379@georgiasouthern.edu).

## Supplementary Materials

The following supporting information can be downloaded at https://www.mdpi.com/article/10.3390/ejihpe16080120/s1. Figure S1: Study level NIH NHLBI risk-of bias traffic-light plot. Table S1: Full-text screening results.

## Abbreviations

The following abbreviations are used in this manuscript:

ABCD	Adolescent Brain Cognitive Development Study
ADHD	Attention-deficit/hyperactivity disorder
BRIEF	Behavior Rating Inventory of Executive Function
BRIEF-2	Behavior Rating Inventory of Executive Function, Second Edition
CBCL	Child Behavior Checklist
CSHQ	Children’s Sleep Habits Questionnaire
DERS-16	Difficulties in Emotion Regulation Scale–16-item version
D-KEFS	Delis–Kaplan Executive Function System
EF	Executive function
EGM	Evidence and gap map
fMRI	Functional magnetic resonance imaging
GPA	Grade point average
MeSH	Medical Subject Headings
NHLBI	National Heart, Lung, and Blood Institute
NIH	National Institutes of Health
PECO	Population–Exposure–Comparator–Outcome
PRISMA	Preferred Reporting Items for Systematic Reviews and Meta-Analyses
PROMIS	Patient-Reported Outcomes Measurement Information System
PROSPERO	International Prospective Register of Systematic Reviews
PSG	Polysomnography
PSQI	Pittsburgh Sleep Quality Index
PVT	Psychomotor Vigilance Test
RCT	Randomized controlled trial
SART	Sustained Attention to Response Task
SDQ	Strengths and Difficulties Questionnaire
SES	Socioeconomic status
UPPS-P	Urgency, Premeditation, Perseverance, Sensation Seeking, and Positive Urgency Impulsive Behavior Scale
WASO	Wake after sleep onset
YSR	Youth Self-Report
