# Supplementary material for: Sleep and the Developing Brain: What We Know, What We Don’t, and Where to Act—A Systematic Review and Evidence and Gap Map
Source: Eur J Investig Health Psychol Educ. 2026 Aug 20;16(8):120. doi: 10.3390/ejihpe16080120 (PMC13512791; doi:10.3390/ejihpe16080120)
Supplement: Supplementary file 1 [file ejihpe-16-00120-s001.zip › ejihpe-4455653-PRISMA checklist.pdf]

PRISMA 2020 Checklist

Section and Topic	Item #	Checklist item	Location where item is reported
**TITLE**			
Title	1	Identify the report as a systematic review.	Title page — identified as a systematic review / evidence and gap map ("Type of the Paper (Systematic Review)"; title: "...A Systematic Review and Evidence and Gap Map").
**ABSTRACT**			
Abstract	2	See the PRISMA 2020 for Abstracts checklist.	Abstract (p. 1).
**INTRODUCTION**			
Rationale	3	Describe the rationale for the review in the context of existing knowledge.	Introduction, Section 1, paragraphs 1–3.
Objectives	4	Provide an explicit statement of the objective(s) or question(s) the review addresses.	Introduction, Section 1, final paragraph ("Accordingly, this systematic review addressed the following question...").
**METHODS**			
Eligibility criteria	5	Specify the inclusion and exclusion criteria for the review and how studies were grouped for the syntheses.	Section 2.2 "Eligibility criteria" (PECO framework: population, exposure, comparator, outcome, study design, inclusion/exclusion criteria); grouping into synthesis domains described in Section 2.6.
Information sources	6	Specify all databases, registers, websites, organisations, reference lists and other sources searched or consulted to identify studies. Specify the date when each source was last searched or consulted.	Section 2.3 "Information sources and search strategy" — PubMed, PsycINFO, Scopus, and Web of Science Core Collection, searched from database inception to May 2026 (no lower date limit).
Search strategy	7	Present the full search strategies for all databases, registers and websites, including any filters and limits used.	Section 2.3 and Table 1 (full search strings and filters/limits for each of the four databases).
Selection process	8	Specify the methods used to decide whether a study met the inclusion criteria of the review, including how many reviewers screened each record and each report retrieved, whether they worked independently, and if applicable, details of automation tools used in the process.	Section 2.4 "Study selection" — records exported to Rayyan; two

PRISMA 2020 Checklist

Section and Topic	Item #	Checklist item	Location where item is reported
			independent reviewers (I.K., M.T.) screened titles/abstracts and full texts; disagreements resolved by discussion/third-reviewer (K.Sh.) adjudication; no automation tools used.
Data collection process	9	Specify the methods used to collect data from reports, including how many reviewers collected data from each report, whether they worked independently, any processes for obtaining or confirming data from study investigators, and if applicable, details of automation tools used in the process.	Section 2.5 "Data extraction" — two independent reviewers (I.K., M.T.) using a piloted structured extraction form; discrepancies resolved through discussion and consensus with a third reviewer (K.Sh.); no automation tools used.
Data items	10a	List and define all outcomes for which data were sought. Specify whether all results that were compatible with each outcome domain in each study were sought (e.g. for all measures, time points, analyses), and if not, the methods used to decide which results to collect.	Sections 2.5–2.6 — outcome domains defined a priori (four developmental outcome domains, Section 2.6); decision rule for selecting the primary/composite estimate when multiple effect sizes were reported for the same association (Section 2.5, item 6).
	10b	List and define all other variables for which data were sought (e.g. participant and intervention characteristics, funding sources). Describe any assumptions made about any missing or unclear information.	Section 2.5, extraction items (1)–(9): bibliographic and study characteristics, sleep exposure definition/measurement, covariates adjusted for, effect moderators, and methodological-quality indicators.
Study risk of bias assessment	11	Specify the methods used to assess risk of bias in the included studies, including details of the tool(s) used, how many reviewers assessed each study and whether they worked independently, and if applicable, details of automation tools used in the process.	Section 2.7 "Risk of bias and methodological quality assessment" — design-appropriate NIH NHLBI Quality

Section and Topic	Item #	Checklist item	Location where item is reported
			Assessment Tools, applied independently by two reviewers with third-reviewer adjudication.
Effect measures	12	Specify for each outcome the effect measure(s) (e.g. risk ratio, mean difference) used in the synthesis or presentation of results.	Not applicable as a pooled meta-analysis: a conventional meta-analysis was not performed (Section 2.8). Individual-study effect measures (Cohen's d, correlation coefficients, odds ratios, 95% CIs where reported) are described in Section 2.5 and presented narratively in Table 2.
Synthesis methods	13a	Describe the processes used to decide which studies were eligible for each synthesis (e.g. tabulating the study intervention characteristics and comparing against the planned groups for each synthesis (item #5)).	Section 2.8 "Data synthesis: evidence and gap map approach" — criteria for cross-tabulating the four sleep-exposure domains against the four outcome domains into the EGM.
	13b	Describe any methods required to prepare the data for presentation or synthesis, such as handling of missing summary statistics, or data conversions.	Not applicable — no pooled meta-analysis or numerical data conversion was performed; the review used an evidence and gap map approach instead (Section 2.8).
	13c	Describe any methods used to tabulate or visually display results of individual studies and syntheses.	Section 2.8 (EGM matrix construction) and Section 3.3; results tabulated/displayed in Table 2 and Figures 2–5.
	13d	Describe any methods used to synthesize results and provide a rationale for the choice(s). If meta-analysis was performed, describe the model(s), method(s) to identify the presence and extent of statistical heterogeneity, and software package(s) used.	Section 2.8 — rationale for using a theory-driven evidence and gap map instead of pooled meta-analysis, given heterogeneity in design, measurement, and effect-size reporting; four-tier cell-level

PRISMA 2020 Checklist

Section and Topic	Item #	Checklist item	Location where item is reported
			evidence-strength classification; analysis/visualization conducted in R v4.5.1 (R Core Team, 2025). No meta-analytic pooling or heterogeneity statistics (e.g., I^2) were computed, as no quantitative synthesis was performed.
	13e	Describe any methods used to explore possible causes of heterogeneity among study results (e.g. subgroup analysis, meta-regression).	Not applicable — no meta-analysis, subgroup analysis, or meta-regression was performed. Domain-level variability in findings is discussed narratively in Section 3.3 and Discussion Section 4.1.
	13f	Describe any sensitivity analyses conducted to assess robustness of the synthesized results.	Not applicable — no sensitivity analyses were conducted, consistent with the non-meta-analytic (EGM) synthesis approach (Section 2.8).
Reporting bias assessment	14	Describe any methods used to assess risk of bias due to missing results in a synthesis (arising from reporting biases).	Not formally assessed.
Certainty assessment	15	Describe any methods used to assess certainty (or confidence) in the body of evidence for an outcome.	Not formally assessed.
**RESULTS**			
Study selection	16a	Describe the results of the search and selection process, from the number of records identified in the search to the number of studies included in the review, ideally using a flow diagram.	Section 3.1 "Study selection" and Figure 1 (PRISMA 2020 flow diagram): 10,437 records identified, 4,150 duplicates removed, 6,287 screened, 643 sought for full text, 25 studies included.
	16b	Cite studies that might appear to meet the inclusion criteria, but which were excluded, and explain why they were excluded.	Section 3.1 — counts and categorized reasons for full-text exclusion (n = 618: clinical/non-normotypic population n = 480; no eligible

PRISMA 2020 Checklist

Section and Topic	Item #	Checklist item	Location where item is reported
			outcome n = 83; non-original design n = 51); 4 additional reports excluded as overlapping cohorts, with citations given.
Study characteristics	17	Cite each included study and present its characteristics.	Section 3.2 "Characteristics of included studies" and Table 2 (all 25 included studies cited, with design, sample, sleep exposure/measurement, outcome domain, main finding, and NIH quality rating).
Risk of bias in studies	18	Present assessments of risk of bias for each included study.	Section 3.4 "Methodological quality of included studies"; per-study ratings in Table 2; Supplemental Figure S1 (item-level traffic-light plot); Figure 6 (common limitations).
Results of individual studies	19	For all outcomes, present, for each study: (a) summary statistics for each group (where appropriate) and (b) an effect estimate and its precision (e.g. confidence/credible interval), ideally using structured tables or plots.	Table 2 (per-study design, sample, exposure/outcome measures, and main finding) and Figure 3 (individual study–outcome effect-direction harvest plot); precision estimates (CIs) not uniformly available across studies and not pooled, consistent with the EGM approach (Section 2.8).
Results of syntheses	20a	For each synthesis, briefly summarise the characteristics and risk of bias among contributing studies.	Section 3.3 "Evidence and gap map analysis" and Figure 2 (evidence-strength map by domain, including contributing-study design and risk-of-bias profile).
	20b	Present results of all statistical syntheses conducted. If meta-analysis was done, present for each the summary estimate and its precision (e.g. confidence/credible interval) and measures of statistical heterogeneity. If comparing groups, describe the direction of	Section 3.3 and Figures 2–4. No statistical

Section and Topic	Item #	Checklist item	Location where item is reported
		the effect.	(pooled) synthesis was conducted, so summary estimates/heterogeneity statistics are not applicable (Section 2.8); direction and consistency of associations are reported instead.
	20c	Present results of all investigations of possible causes of heterogeneity among study results.	Not applicable — no formal investigation of heterogeneity (e.g., subgroup analysis) was conducted; qualitative discussion of variability by domain appears in Section 3.3 and Discussion Section 4.1.
	20d	Present results of all sensitivity analyses conducted to assess the robustness of the synthesized results.	Not applicable — no sensitivity analyses were conducted.
Reporting biases	21	Present assessments of risk of bias due to missing results (arising from reporting biases) for each synthesis assessed.	Not formally assessed.
Certainty of evidence	22	Present assessments of certainty (or confidence) in the body of evidence for each outcome assessed.	Not formally assessed.
**DISCUSSION**			
Discussion	23a	Provide a general interpretation of the results in the context of other evidence.	Section 4 "Discussion" and 4.1 "Interpreting the pattern in light of current evidence" (results contextualized against prior reviews and mechanistic/experimental literature).
	23b	Discuss any limitations of the evidence included in the review.	Section 5 "Conclusions and Limitations", paragraph 2 (evidentiary limitations: heterogeneity precluding meta-analysis, predominance of cross-sectional/observational designs outside the duration domain, residual confounding).
	23c	Discuss any limitations of the review processes used.	Section 5 "Conclusions and Limitations", paragraph 2 (review-

Section and Topic	Item #	Checklist item	Location where item is reported
			process limitations: restriction to four databases without citation-chasing/hand-searching, English-language/peer-reviewed-only criteria, reliance on a priori evidence-tier definitions rather than an external validated scale, non-independence of cell counts since study-outcome links were the synthesis unit).
	23d	Discuss implications of the results for practice, policy, and future research.	Section 4.2 "Practical implications" and Section 5 (implications for practice/policy — e.g., school start-time reform, multidimensional sleep-health screening — and for future research, e.g., napping in younger children, mechanistic and longitudinal studies of timing/regularity and sleepiness).
**OTHER INFORMATION**			
Registration and protocol	24a	Provide registration information for the review, including register name and registration number, or state that the review was not registered.	Registered prospectively on PROSPERO (International Prospective Register of Systematic Reviews; ID: CRD420261438604), stated in Section 2.1.
	24b	Indicate where the review protocol can be accessed, or state that a protocol was not prepared.	Section 2.1; the registered protocol is accessible via the PROSPERO record (ID: CRD420261438604). No separate protocol repository (e.g., OSF) is cited in the manuscript.
	24c	Describe and explain any amendments to information provided at registration or in the protocol.	Not reported — the manuscript does not describe any

Section and Topic	Item #	Checklist item	Location where item is reported
			amendments to the registered protocol.
Support	25	Describe sources of financial or non-financial support for the review, and the role of the funders or sponsors in the review.	"Funding" statement (end matter): "This research received no external funding."
Competing interests	26	Declare any competing interests of review authors.	"Conflicts of Interest" statement (end matter): the authors declare no conflicts of interest.
Availability of data, code and other materials	27	Report which of the following are publicly available and where they can be found: template data collection forms; data extracted from included studies; data used for all analyses; analytic code; any other materials used in the review.	"Data Availability Statement" (end matter): original contributions are included in the article; further inquiries may be directed to the corresponding author. No separate extraction-form, dataset, or code repository is cited.

From: Page MJ, McKenzie JE, Bossuyt PM, Boutron I, Hoffmann TC, Mulrow CD, et al. The PRISMA 2020 statement: an updated guideline for reporting systematic reviews. BMJ 2021;372:n71. doi: 10.1136/bmj.n71.
This work is licensed under CC BY 4.0. To view a copy of this license, visit https://creativecommons.org/licenses/by/4.0/
